# Older people’s perceptions and experiences of older people with the Sit-to-stand activity: An ethnographic pre-feasibility study

**DOI:** 10.1590/1518-8345.6128.3813

**Published:** 2023-01-30

**Authors:** Uirá Duarte Wisnesky, Joanne Olson, Pauline Paul, Sherry Dahlke

**Affiliations:** 1 University of Alberta, Faculty of Medicine and Dentistry, Edmonton, Alberta, Canada.; 2 University of Alberta, Faculty of Nursing, Edmonton, Alberta, Canada.; 3 Scholarship holder at the Conselho Nacional de Desenvolvimento Científico e Tecnológico (CNPq), Brazil

**Keywords:** Aged, Mobility Limitation, Feasibility Studies, Ethnography, Qualitative Research, Brazil, Idoso, Limitação da Mobilidade, Estudos de Viabilidade, Etnografia, Pesquisa Qualitativa, Brasil, Anciano, Limitación de la Movilidad, Estudios de Factibilidad, Etnografía, Investigación Cualitativa, Brasil

## Abstract

**Objective:**

the purpose of this pre-feasibility study was to examine perceptions and experiences of a Sit-to-stand activity with urban Brazilian community-dwelling older people in their homes.

**Method:**

the exploration method was focused ethnography. Purposive sampling was used to recruit 20 older people. Five means of data generation were used, namely: socio-demographic surveys, participant observations, informal interviews, formal semi-structured interviews, and field notes. Data analysis was qualitative content analysis.

**Results:**

the experience of mobility-challenged older people with the Sit-to-stand activity was dependent on their mobility expectations involving many factors that worked together to influence their beliefs and attitudes towards the activity, preferences, behaviors, and cultural perceptions. The participants of this study seemed to find the activity enjoyable; however, the most noticeable shortcomings for their engagement in the Sit-to-stand activity emerged as gaps in their personal and intrapersonal needs.

**Conclusion:**

the recommendations generated from the study findings call for the design of implementation strategies for the Sit-to-stand intervention that are tailored to this particular population’s needs.

## Resumo

**Objetivo:**

o objetivo deste estudo de pré-viabilidade foi examinar percepções e experiências da atividade de *Sit-to-stand* com idosos brasileiros residentes em suas casas, no meio urbano.

**Método:**

o método exploratório foi etnografia focada. Foi utilizada a amostragem intencional para recrutar 20 idosos. Foram utilizados cinco meios de geração de dados: inquéritos sociodemográficos, observações participantes, entrevistas informais, entrevistas formais semiestruturadas e notas de campo. Os dados foram analisados mediante análise de conteúdo qualitativo.

**Resultados:**

a experiência dos idosos com problemas de mobilidade na realização da atividade *Sit-to-stand* dependia de suas expectativas de mobilidade envolvendo muitos fatores coordenados que, de forma conjunta, influenciaram suas crenças e atitudes em relação à atividade, suas preferências, seus comportamentos e percepções culturais. Os participantes deste estudo pareciam considerar a atividade aprazível; no entanto, as deficiências mais perceptíveis para o engajamento dos participantes na atividade *Sit-to-stand* surgiram de falhas em suas necessidades pessoais e intrapessoais.

**Conclusão:**

as recomendações geradas a partir dos achados do estudo convocam a concepção de estratégias de implementação da intervenção *Sit-to-stand* adaptadas às necessidades dessa população em particular.

## Resumen

**Objetivo:**

el propósito de este estudio de viabilidad previa fue examinar percepciones y experiencias con respecto a la actividad *Sit-to-stand* entre los adultos mayores de Brasil que viven en sus hogares en comunidades urbanas.

**Método:**

el método de exploración se enfocó en la etnografía. Se utilizó muestreo intencional para reclutar 20 adultos mayores. Se emplearon cinco medios para generar datos, a saber: encuestas sociodemográficas, observaciones participantes, entrevistas informales, entrevistas formales semiestructuradas y notas de campo. Para el análisis de los datos se recurrió a análisis de contenido cualitativo.

**Resultados:**

la experiencia de los adultos mayores con problemas de movilidad en relación con la actividad *Sit-to-stand* dependió de sus expectativas en torno a la movilidad, las cuales implicaron muchos factores que actuaron en conjunto para influenciar sus creencias y actitudes con respecto a la actividad, al igual que preferencias, conductas y percepciones culturales. Aparentemente, a los participantes de este estudio la actividad les resultó amena; sin embargo, los inconvenientes más notorios para adoptar la actividad *Sit-to-stand* surgió en la forma de déficits en sus necesidades personales e intrapersonales.

**Conclusión:**

las recomendaciones resultantes de los hallazgos del estudio indican la necesidad de diseñar estrategias de implementación para la intervención *Sit-to-stand* a la medida de las necesidades de este grupo poblacional específico.

**Highlights**

(1) Care of older people should also be considered from the cultural context.

(2) Care plans for older people must consider their experiences.

(3) Nurses need to support older people to maintain their independence.

(4) This study contributes to understanding reasons for engagement with the intervention

## Introduction

Increased longevity is part of the demographic transition in most countries and Brazil is no exception. The aging process draws attention to the health conditions of older people because this phenomenon is accompanied by a higher risk of disability and morbidities^1^. As people age, some physiological, psychological and social changes occur^2^ ^-^ ^3^. These changes can lead to a variety of syndromes and issues, which, in turn, can lead to poor health outcomes^4^. For instance, critical illnesses and chronic diseases carry substantial risk of decreased functional capacity for older people^5^. In addition, functional inability can worsen already poor health outcomes.

Functional capacity is a relevant indicator of health status in older people and it is closely related to quality of life^6^. Functional capacity and inability are seen in terms of maintenance of autonomy, which for older people can be conceptualized as the ability to perform self-care, self-maintenance and physical activity^7^. Thus, mobility challenges may indicate functional decline and is a relevant problem for many older people.

Simply defined as the ability to move safely from one place to another, mobility is fundamental for engaging in daily activities^8^ and allowing older people to lead independent lives. A well-known framework described five interrelated components of mobility: physical, cognitive, psychosocial, environmental, and financial domains^9^. In this framework, mobility is “the ability to move within community environments that expand from one’s own home, to the neighborhood, and to regions beyond”. We draw from a similar framework to underpin this study, the International Classification of Functioning, Disability and Health^10^, which states that mobility influences and is influenced by determinants within different dimensions, such as health conditions, body functions and structures, participation, and contextual factors (i.e., environmental and personal factors). Both frameworks capture indoor and outdoor movement as well as the use of assistive devices and transportation^9^ ^-^ ^10^. However, the International Classification of Functioning, Disability and Health^10^ framework emphasizes participation in activities as a pivotal dimension, for it plays a major role in mobility. In addition, the International Classification of Functioning, Disability and Health^10^ framework supports contextual factors as strong determinants, either as facilitators or barriers, of functional outcomes. These include external environmental factors such as community, socioeconomic status and access to health care, as well as personal factors such as demographic characteristics, culture and upbringing, lifestyle preferences, motivation and personality traits.

The prevalence of mobility challenges among older people in Brazil is high. According to researchers’ findings, mobility challenges affect 15% and 24% of people aged 60 and over and 70 years old and over, respectively^11^. This is corroborated by a survey conducted in 2013 in Brazil measuring mobility through a hierarchical approach using activities of daily living^12^. With regard to performing basic and instrumental activities of daily living, 30.1% of the Brazilian older people (60 years old or over) reported having difficulty. In addition, prevalence tends to surge with age - 16.4% among the older people aged 60-64 years old reported facing challenges to perform activities of daily living, when compared to 48.3% among those aged 75 and over^12^.

In terms of global guidelines, mobility has been highlighted as a goal for healthy aging - the process of developing and maintaining the functional capacity that allows well-being in older age^13^. For instance, the United Nations Decade of Healthy Ageing (2021-2030)^13^ and the Sustainable Development Goals^14^ bring together governments, civil society, international agencies, professionals, academia, the media, and the private sector to improve the lives of older people, their families, and the communities in which they live to promote older people’s mobility, which is paramount to functional capacity^15^.

In practice, a wide range of interventions have been developed to promote mobility. Special attention is given to interventions founded on the principle that some critical predictors of mobility decline are modifiable^16^. Therefore, physical activity emerges as a possible intervention to improve or maintain mobility in older people by remodeling critical determinants^17^ ^-^ ^18^. There is evidence that mobility-challenged older people benefit from interventions targeting muscular strength, flexibility and balance^19^, such as the Sit-to-stand activity.

The Sit-to-stand motion is a transition movement and one of the most frequently performed by humans^20^. Rising from a sitting to a standing position is considered a prerequisite to walking and other functional activities. The Sit-to-stand activity is a simple action involving the act of repeatedly sitting down and standing up from a chair, and is frequently completed in the context of daily life. Given the relevance of the ability to rise from a seated to a standing position and vice-versa, the Sit-to-stand activity emerges as a likely intervention to effectively improve or maintain mobility in older people. This is corroborated by studies from countries in the Global North suggesting that repeated Sit-to-stand activity can maintain or improve mobility in older people^21^.

While some of the benefits and advantages of the Sit-to-stand intervention have been well documented, most of this research has been conducted in countries from the Global North. This makes identification of the impact of modifiable determinants (barriers and enablers) for engaging in the Sit-to-stand activity not clear for people from the Global South, which, in turn, makes it more difficult to implement those interventions in these settings^22^ ^-^ ^24^. Therefore, appropriate and effective interventions to improve mobility are not reaching many of those in need. Rarely does knowledge translation of interventions prioritize the individuals, nations or communities who most need the knowledge, which maintains gaps in health literacy and inequalities between the researchers and the researched^25^. Within the production of knowledge systems, research without practical relevance or application has no merit or value. Our study contributes to the relevance of taking into consideration the contextual factors during the knowledge translation process.

All things considered, it is not enough to solely transfer successful interventions from one country or setting to another. Before conducting an intervention study, it is important to first examine the context wherein the intervention would be introduced^24^. Particularly for Brazilian community-dwelling older people living with mobility challenges, mobility involves cultural elements; it is an integral part of their sense of self and is strongly related to contextual factors^26^. In line with a knowledge translation standpoint, this pre-feasibility research is the first step to link the Sit-to-stand activity, a successful intervention from the Global North, and culturally relevant knowledge produced during the research to what Brazilian older people know about mobility and physical activity. The objective of this pre-feasibility study was to examine perceptions and experiences of a Sit-to-stand activity with urban Brazilian community-dwelling older people in their homes.

## Method

### Study design

Many factors can affect the successful implementation and validity of an intervention. Therefore, the primary purpose of employing a pre-feasibility (and feasibility) approach is to assess the prospects for successful implementation of a potential intervention and to reduce threats to the validity of these interventions. Focused ethnography was the qualitative approach selected to examine urban Brazilian community-dwelling older peoples experiences of a Sit-to-stand activity^27^. Constructivism was the assumption underpinning this study, which adhered to the Consolidated Criteria on Reporting Qualitative Research - COREQ^28^.

### Setting and participants

The Family Health Strategy unit at a healthcare center located in Paquetá, an island in Rio de Janeiro, Brazil, was selected as the study setting. In Brazil, a Family Health Strategy unit delivers primary health care through the public health system. It has multidisciplinary teams, including community health workers, which are responsible for meeting the heath care needs of approximately 1,000 households in a defined geographical area.

After access was granted at the Family Health Strategy unit and potential participants were identified, healthcare providers from the health center acted as gatekeepers and asked the participants if they agreed to put their contact details on a list so that they could be approached to take part in a health research study. The potential participants were contacted by the first author (who was a nurse with a master’s degree in both social sciences and history and was a PhD candidate in nursing at the time of the research) to receive more details about the study, as well as to discuss reasons for doing the research.

Purposive sampling was employed to select the participants^29^. Our anticipated final sample size was 20 participants, as this was consistent with the mean sample sizes of two studies of exercise/physical activity with an older population using focused ethnography^30^ ^-^ ^31^. Sampling was only discontinued when we achieved informational redundancy^32^.

Older people were included in the study if they: (1) had a self-reported mobility challenge such as difficulty with mobility when performing any basic or instrumental activities of daily living, or were unable to engage in social activities; (2) lived in the community; (3) were ≥ 60 years of age; (4) were able to understand and communicate in Portuguese; (5) were able to provide their written informed consent in Portuguese; and (6) did not present any pre-existing diagnosis of cognitive challenge and were considered by their healthcare providers as having sound cognitive competence, which was based on their health history and on the care provider’s judgment. The exclusion criteria for older people were as follows: (1) self-reported inability to Sit-to-stand; and (2) the researcher observing the participant’s inability to sit/stand up.

### Procedures

Ethical approval was granted by the Institutional Review Board at the university where the authors are affiliated (#Pro00081957) and in the country where the study took place (#2,878,399 and #CAAE 95969318.4.0000.5650). The data were generated during home visits. Five means of data generation were used, namely: socio-demographic surveys, participant observations, informal interviews, formal semi-structured interviews, and field notes. The first author performed the observations and interviews. After the participants gave their verbal consent to participate in the study, six home visits were planned with each of them. In the first home visit, the study information was reviewed with the participants and their written informed consent was obtained. Subsequently, the researcher asked them to answer the socio-demographic survey. At the end of the first visit, the researcher taught the Sit-to-stand activity to the participants. 

From the second to the fifth home visit, the researcher perform participant observations and informal interviews as activities to generate data. Once a week and for four weeks, the researcher observed how the participants performed the Sit-to-stand activity and conducted informal interviews on topics regarding their experiences and perceptions in relation to the Sit-to-stand activity. We pre-established this 4-week timeframe to perform participant observations and informal interviews because we believed that a careful sampling strategy and detailed field notes, combined with the researcher’s reflexive approach, would be sufficient to observe both the participants’ mobility patterns and their Sit-to-stand performance. Nonetheless, the data generation activities were only to be discontinued when informational redundancy was achieved.

As for the informal ethnographic interview, “[it] occurs whenever the researcher asks someone a question during the course of participant observation”^33^. The focus of these informal interviews was to allow the researcher to engage with the participants while they went about their daily activities and convey information in culturally patterned ways. During these informal interviews, the researcher mainly asked questions to expand on her field observations.

Finally, the sixth and last visit was devoted to the formal interview focused on the research question: How do Brazilian older people living with mobility challenges experience the Sit-to-stand activity? The last visit was also devoted to the “getting out” or disengagement phase, where the researcher went over the study pre-established goals and accomplishments (e.g., objectives, timelines). This phase ended with an offer to send a report of the research findings to the participants, upon completion^34^. None of the authors were known to the participants before the study. The data were generated between June and October 2018.

The home visits occurred according to the participants’ availability in the morning or afternoon. The observations were made employing purpose-built observational logs. These logs included the following topics related to the participants’ mobility: noting the arrangement of their physical space; presence and arrangement of individuals within that space; and noting the individuals’ specific activities and movement.

For the formal interview, a semi-structured guide was used (Figure 1). These questions were developed collaboratively by all members of the research team, and piloted prior to the study with three individuals who shared similar characteristics to the group of participants included in this study to ensure clear understanding of the questions. None of the individuals who piloted the interview questions took part in the actual study. After piloting the interview questions, changes were made to the guiding questions used.

**Figure 1:** Interview guide for perceptions and experiences of a Sit-to-stand activity with urban Brazilian community-dwelling older people. Rio de Janeiro, RJ, Brazil, 2018

Questions
Can you tell me using as many details as possible about the Sit-to-stand activity you have performed during the last 4 weeks?
Did you talk to any community members about positive or negative effects of the activity? If yes, can you share with me some of the content of your discussions?
How do you feel in relation to your mobility after finishing the activity?
Do you intend to keep doing the activity after the study ends?
Do you have any comments on the content and design of the Sit-to-stand activity? Anything we could do better? Anything that would encourage you to do it?
These are all my questions for this interview. Would you like to add anything, or say something that you think is important but it was not discussed, before we end this interview?

The interviewer facilitated all discussions in Portuguese, which is the language spoken by the participants and her own mother tongue. At times, upon the participants’ request, their family members were present during data generation; however, these family members did not answer for the participants and were not included in the research process. The interviews were audio-recorded and field notes were made after the observations and informal interviews. When necessary, these notes were used to clarify any inaudible recordings, to consider the Sit-to-stand activity patterns performed by the participants, and to better promote reflection during data analysis. The passive to moderate observation^33^ sessions lasted a mean of 15-20 minutes *per* participant and were performed during the six encounters, totaling a mean of 33 observation hours. Each formal semi-structured interview lasted from 30 minutes to one hour. In order to establish and maintain responsible “data stewardship” practices, all components of the ethnographic record were digitalized and encrypted. The data were stored, managed, accessed and analyzed within a secured SharePoint environment.

Member checking was performed simultaneously during each interview when the interviewer summarized what was said and asked the participants whether their understanding was realistic, fair and representative in order to minimize distortion of ﬁndings. Member checking was then employed as a reflexive mode of knowledge production^35^. It ensured agreement in the representation, as the participants had the opportunity to discuss and negotiate the meanings during the interviews.

### Data analysis

All the audio-recordings from interviews were transcribed by the first author. Data analysis was an iterative process performed simultaneously with data generation. Data translation was performed (Portuguese and English) because the study participants and some of the researchers spoke different languages. The data translation procedures are described elsewhere^26^. The transcripts were imported to the NVivo 11 data management software (QSR International Pty Ltd, Victoria, Australia). A qualitative content analysis was inductively performed and key themes were generated. The steps that followed were (1) coding and categorization; (2) memoing; and (3) theming^36^.

Coding was initiated by re-reading the transcripts to become more familiar with the data and to obtain a sense of the whole. Subsequently, key words and phrases were highlighted line-by-line with analytic notes recorded in the margins. Then, when important words and phrases were identified and labeled, codes were created based on words and ideas within the data (also known as open coding). At first, codes were broad and generic to help us make sense of the data, and more abstract codes were developed as the analysis progressed. With each new concept that appeared, previous data were reviewed to add additional codes where appropriate.

As the coding process continued, categories were created, linking together the relationships between codes and ideas within the data. To assist in the development of categorization, the data were reorganized by grouping together related codes rather than by chronological order. Using NVivo 11, a data matrix was generated to summarize the data and to compare and contrast the experiences of each participant. The final categories were the ones that re-occurred frequently in the interviews and/or were central concepts in answering the research questions. A summary was then written for each category, which was then reviewed for homogeneity.

Themes were built from relationships between the codes, categories, and the larger patterns woven throughout the data that made up the “cultural scene”^37^. With an ongoing analysis of the interviews, developing themes were incorporated into follow-up interview questions. This helped us identify patterns among participants and consider or reject generated concepts.

During this entire process, analytic memos were formulated. They were free-style records of the ideas or insights the researcher had about the data. This strategy was used to assist the researcher in making conceptual leaps from raw data to those abstractions that explain research phenomena in the context in which they were examined. The analysis was initiated and led by the first author, and further analyzed and discussed among all authors until consensus was reached to increase reliability of the findings. The participants were assigned pseudonyms for de-identification.

### Reliability

Rigor is characterized when the research methods are justified, the process is transparent, the outcomes are defendable, and the findings are viewed as applicable by research consumers. For this study, we borrowed Tracy’s^38^ criteria for operationalizing reliability of qualitative data. Throughout the article we showed that this study (1) is about a worthy topic that is relevant, timely, significant and interesting, as well as it makes a unique contribution to the health field; (2) has resonance by means of transferability, which is the potential of the study to be relevant in other contexts and with other populations; (3) makes a significant contribution by generating further debate and inspiring future research, and (4) possesses ethical integrity, which was attempted by addressing ethical requirements and avoiding deductive disclosure, i.e., identification of an individual’s identity using known characteristics of that person.

In addition, to achieve sincerity, the first author wrote a reflexive journal, where she logged the details of how her assumptions and personal reactions may have influenced the results of each interview and observation, her agenda as a researcher, her assumptions as an individual, her research process, the intersubjectivity of the findings, and self-interrogations. In addition, the first author kept an audit trail to track her choices, hunches and interpretations. Equally important for credibility, accuracy of representation was attempted through crystallization of meanings between the authors. According to Tracy^38^, “the goal [of crystallization] is not to provide researchers with a more valid singular truth, but to open up a more complex, in-depth, but still thoroughly partial, understanding of the issue”.

## Results

The list of potential participants who consented to be approached included 51 individuals. When data generation was initiated, five of the 51 potential participants had passed away, four refused to participate, three had moved, sixteen were not eligible to participate by the time we approached them due to health conditions, and three refused to participate stating inability due to physical decline. In the end, 20 participants finished the 4-week Sit-to-stand activity. Figure 2 shows characteristics of the 20 individuals who participated in this study.

**Figure 2:** Demographic data of urban Brazilian community-dwelling older people. Rio de Janeiro, RJ, Brazil, 2018

Pseudonym	Biological sex	Age	Ethnicity	Marital status	Lives alone	Number of people living in the same household*	With whom they live	Use of mobility device	Need help with the activity	Religion
Acacia	Female	89	White	Widowed	No	2	Sons	Yes	Yes	Catholic
Luz	Female	60	Brown	Single	No	1	Niece	Yes	Yes	Spiritism
Florian	Male	81	Black	Widowed	No	2	Daughters	Yes	No	Catholic
Butterfly	Female	68	White	Widowed	No	1	Daughter	No	No	Pentecostal
Oak	Male	89	White	Married	No	5	Wife and children	No	Yes	Pentecostal
Fox	Male	75	White	Married	No	3	Wife and children	Yes	No	Pentecostal
Juliette	Female	88	Black	Widowed	No	3	Daughter and grandchildren	Yes	Yes	Catholic
Lily	Female	98	White	Widowed	No	1	Daughter	No	Yes	Catholic
Rose	Female	97	White	Widowed	No	1	Daughter	No	Yes	Spiritism
Carnation	Female	77	Brown	Widowed	No	1	Brother	Yes	Yes	Pentecostal
Lavender	Female	83	Black	Married	No	2	Husband and son	Yes	Yes	Umbanda
Daisy	Female	74	Brown	Divorced	No	2	Son and daughter-in-law	No	No	Not declared
Sky	Female	71	Brown	Single	No	1	Son	Yes	Yes	Catholic
Petal	Female	83	White	Widowed	No	2	Caregiver	Yes	No	Catholic
Flora	Female	81	White	Widowed	Yes	0	-	Yes	Yes	Catholic
Ella	Female	82	White	Widowed	No	3	Daughter, son-in-law and grandchild	No	No	Catholic
Eva	Female	70	Brown	Widowed	No	2	Sons	Yes	Yes	Spiritism
Jade	Female	83	White	Widowed	No	2	Son and daughter-in-law	Yes	Yes	Pentecostal
Spring	Female	87	White	Widowed	No	3	Daughter, son-in-law and grandchild	No	No	Catholic
Sonata	Female	93	White	Single	No	1	Daughter	Yes	Yes	Catholic

* Excluding the participant

This section presents the analysis of the data from the participant observations and interviews. Two themes were constructed during the analysis. The first theme, “to join, or not to join: that is the question”, started to be elaborated after the first home visit, where the first author introduced the Sit-to-stand activity to the participants. If the participants were curious about the activity, the researcher asked them about their first impressions of the activity. The researcher paid attention to the participants’ initial reactions after introducing the activity. She wondered about which aspects of the activity were of interest to the participants at the outset as she tried to understand their motivation and reasons to join, or not, the Sit-to-stand activity. The subthemes related to the participants’ first impressions of the Sit-to-stand activity were as follows: “structural aspects” and “individual influence”. The categories linked to the “structural aspects” subtheme were the following: “having a flexible schedule”, “trying something new”, “changing the routine”, and “having someone to do it with”. The categories linked to the “individual influence” subtheme were “having physical limitations”, “stigmatization associated with exercises targeting older people”, “establishing intentions” and “having goals”.

The second theme, “how did it go?”, was elaborated during the weekly follow-up visits. The participants and the researcher talked about the Sit-to-stand activity, their progress, their impressions of it, and if there were any unexpected events from the previous week. In the formal and informal interviews, the researcher was interested in knowing the participants’ experiences with the Sit-to-stand activity. Thus, the last formal interview served as an opportunity to summarize and debrief with the participants about progression of the 4-week activity. This interview was also an opportunity for the researcher to take her hunches and ideas back to the participants so they could discuss and negotiate meanings. The subthemes related to their completion of the 4-week activity were “drivers” and “obstacles”. The categories linked to the “drivers” subtheme were as follows: “cost”, “privacy”, “perceived benefits in mental and physical health”, “going at own pace and adapting the activities”, “sense of achievement”, “having a supportive system” and “easily integrated into the daily routine”. The categories linked to the “obstacles” subtheme were the following: “present health condition”, “no perceived need”, “fatalism”, “fear of worse outcome”, “lack of companion”, “lack of motivation”, “competing demands”, “routine challenge/discipline”, “safety concern” and “weather”. Each one of the themes, as well as their respective subthemes and categories, are described in Figure 3 with supporting excerpts from the transcripts and field notes of the interviews with the participants.

**Figure 3:** Themes and excerpts of perceptions and experiences of a Sit-to-stand activity with urban Brazilian community-dwelling older people. Rio de Janeiro, RJ, Brazil, 2018

Theme	Subthemes	Categories	Excerpts
To join, or not to join: that is the question	Structural aspects	Having a flexible schedule	*I think it won’t be hard to do this exercise. You said once a day for a couple of minutes? It seems doable. I can do it after lunch, or maybe after breakfast. Let’s see. Let’s see. Sometimes we join the gym but it’s not easy to show up every time. Something always comes up and at the end, each day I’m going less and less. Maybe with this exercise at home it will be easier* (Ella).
		Trying something new	*If it is to help, I’ll do it. I’ll challenge myself with it, that’s it. I feel a lot of back pain, but I’ll try it out. I tried many different things throughout the years, different drugs, compression socks, I even went to the physiotherapist a couple of times. To be honest, my life is pretty much the same boredom now. Before I used to do everything, up and down. So, it’s always good to try new things, it helps* (Sonata).
		Changing the routine	Florian: *I already have my things, things I already do during the day. I’m not sure if I like it* [the activity]*. I don’t need it in my life.*
			Interviewer: *How come? It would take just five minutes of your time. You could do it after you wake up, before taking a shower.*
			Florian: *I’m not sure, I have my life on a schedule, I like to do my stuff, I already organized my life with other things*
		Having someone to do it with	*I’m afraid, I’m afraid of falling down and breaking my femur again. I need someone to do it with me. I don’t feel well, it’s been a while since I used the walker; my son carries me around. Before he leaves for work, he puts it in the living room. Maybe my son can help me after he gets home from work. If you explain that to him, he’ll do it with me. It’s been a while since I used the walker, I don’t feel safe doing it by myself* (Eva).
	Individual influence	Having physical limitations	*I’m not sure about this exercise. The thing is my leg. I have a lot of pain, a lot of pain, my dear, on the knee. It hurts when I move, when I get up, when I get down because of this arthrosis, very high arthrosis. I still feel a little pain from the varicose veins. The varicose veins hurt a little. When the weather warms up, they start to heat up, to burn. It’s hard for me. I feel like I just can’t, what if I stagger and fall?* (Rose)
		Stigmatization associated with exercises targeting older people	*But this [Sit-to-stand activity] is for people who don’t walk or anything, it’s for seniors or people who are already very sick. Thank you very much, but I don’t want to take your time. This is not for me. I’m old but that is not for me, that’s enough. I’m 80, but I’m still OK* (Summer).
		Establishing intentions	*I’ll try to do [physical activities] as the doctor of the Family Health Strategy asks me to do so. And the other doctors also say: “you have to walk, walking is good, your muscles will thank you, you’ll see”. So, I think I can start with this exercise that you said* (Acacia).
		Having goals	*Oh, I really want to be able to sit down and get up without pain, without problems. Sometimes when I sit down, get up, sit down, get up, my leg pops. But I already felt it before, it’s been a while [that I feel the pop]. I think it keeps popping, for now ... But like, when I go to the bathroom, to climb the step there in the bathroom, I say: “oh, my knee popped. I heard a crack”. But this is normal. The thing is to do the exercise as you said. Better days will come, right. Sit down, get up, sit down again, get up, right? I have to look forward to something, hold on to something and move on* (Juliette).
How did it go?	Drivers	Cost	*You know, I enjoyed doing it, I did it every time I could and every time I remembered. Sometimes I forget to do it and when I remember it’s already bed time. But the thing of just using the chair was pretty nice. If I go to the gym classes on the beach, I have to pay. If I go there in the health center’s gym, I don’t have to pay but to get there I have to spend money on the rickshaw [transportation means]. But that sitting and standing activity can be done at home, that’s cool. I do it at home* (Star).
		Privacy	*You don’t have to get dressed and leave the house. I even do it wearing my old and ripped clothes. People look at you when you’re there at the gym. It’s easier to exercise because you don’t have to worry about clothes, getting dressed, and all that. When I’m at home, it’s just me* (Ella).
		Perceived benefits in mental and physical health	Sonata: *This week wasn’t easy because sometimes I feel this pain in the morning and at night. During the day, the pain alleviates a little because I also have a lot to do. Oh my God. So, I have to do it according to what I can, I’m doing something.*
			Interviewer: *Did you notice any benefit from exercising?*
			Sonata: *Oh yes. Because I didn’t get up from the chair easily. I had to hold it here. If I sat on this stool, it was difficult to get up. So, I had to help myself up with both my hands on the chair, and sometimes it would take longer because of the pain. My whole body hurts. Oh, but my daughter was so happy. I surprised her, right? She got home, because I always have to wait for her to help me. Then she came and I said, “stay here close to me, close your eyes”. Then she said: “what happened?” I said, “a very good thing happened”. She said, “was the girl [researcher] here?” I said, “she was”. “What happened?” She asked. I said, “wait”. Then I said, “you can turn around”. Then I got up by myself. “Huh? You didn’t hold there”, she said. And then I came and did it once, twice, sometimes up to three times, I could not stand up. It wasn’t easy, but thank God, I’m getting there.*
		Going at own pace and adapting the activities	*The exercise is easy. I’m going, I’m going. I’m doing. Once in a while, it’s not easy to get up, okay. I can sit down... I go slowly and surely. I really wanted to sit without pain, right? And sometimes, sometimes it hurts more, others it hurts less, right? The exercise is actually good. It’s easy. It’s not unpleasant, no. Every day I do a little. I sit there in that chair. Yesterday I did it. Sit down, get up, sit down, get up. Sometimes I need help from my daughter or my son-in-law to do it, and I also use my hands to get up. But I do it. When I don’t feel like doing it in that chair, I do it here on the sofa. But I do it. The important thing is that I’m trying* (Juliette).
How did it go?	Drivers	Sense of achievement	Interviewer: *Have you noticed any benefit after having done this exercise? What do you notice about yourself? Are you walking a little more?*
			Sky: *No, not walking yet. Now I’m going to start walking here. I’m not walking yet. I just stand, but I’m already stretching more. After you got the walker for me I started doing the sit and stand thing. I tried and because I felt safe doing this, I do it every day. I’m already stretching further. I believe I’ll improve more. I couldn’t even stretch like that. One step at a tim*e
		Having a supportive system in family members	Interviewer: *Carnation, what would encourage you to do more of this activity? Is your son pushing you?*
			Carnation: *My son ain’t easy, when he sees me he says: “you have to do this and that’s it”. Sometimes, when he walks by and I’m hanging outside, he says, “you’re going out, at last, huh. Way to go”. I say, “fine, fine”. Then he gets all happy, then I do things when he’s around. But when he ain’t around, I don’t do anything*.
			Interviewer: *So he encourages you to exercise more?*
			Carnation: *Very much, he wants me to go to the gym. What am I going to do at the gym? No gym. My son supports me. I’m the one not taking it seriously, it’s just that, because that exercise was a cool thing, but practically everything for health is a good thing. I even talked to my daughter, she said, “mom, that’s right, do the exercise. Are you doing it?” Every now and then I don’t do it, and my daughter says, “mom, you have to do it every day, every time”. Not every time, I can’t. But that [the Sit-to-stand activity] was good for me, to cheer me up. My family supports me*.
		Having a supportive system in the healthcare system	*Last week the girls [nurses] were here, they came here to change the dressing on my leg. It went well. I really like them, because they’re very cool. They changed the dressing. They do it very well. They’re adorable. To tell you the truth, I had a big callus, the size of my toe, here. Then I asked the nurse to clean it a little bit. Oh, she took the scissors, cut and cleaned it. I don’t feel anything anymore. But it was a big callus and I couldn’t even put my shoes on or walk straight. It was a miracle, wasn’t it? She cut here and cleaned it. She passed a lot of medication. It’s dry. She cut everything. I don’t know if she cut it with a razor or with scissors. But it hurt a lot before. I put my foot on the floor and it hurt. After they cleaned everything and took off the callus, it stopped hurting, so I was more comfortable to do this sit and stand thing* (Juliette).
		Easily integrated into the daily routine	Interviewer: *Tell me about the activity, how did it go, how did you do it?*
			Carnation: *I did it and went for a walk. What I did, I always take a morning walk, so as not to forget to do this stand up and sit down thing, I do it before my walk. I get up feeling so lazy, but I get up. I drink my coffee, I do the thing, then, I go for a walk. It was easy to do it [Sit-to-stand activity] because I do it before my walk, but if I don’t walk I forget to do it.*
	Obstacles	Presenting a health condition	*I had a hard time last Thursday. I got sick, I was sick like never before. I felt a lot of pain, I had no strength for anything, nothing. I no longer had [strength] but I got even worse. I went to bed and I couldn’t sleep, I was short of breath. I was without strength for anything. I no longer had, now you see, I underwent seven surgeries, and I have scoliosis. I couldn’t do anything last week. The doctor told me that Tandrilax that takes away my pain when it’s too bad for me. So yesterday I didn’t take Tandrilax, nor today, so I’m in more pain, more in the knee, old folks you know, in the spine, on the shoulders. I can’t even think about doing what you asked me with all this, not even think about it* (Acacia).
		No perceived need	Interviewer: *And what motivates you to exercise? Or what would better motivate you to do the Sit-to-stand activity?*
			Florian: *I don’t feel pain in the knee, I feel nothing. I don’t feel anything, I have this thing in the knee, I walk with a cane; but I don’t feel pain. The knee is almost normal. I don’t need to do anything else, right? I’m in this phase where I don’t feel pain, I don’t feel anything, the only thing I do is walk in the house, I don’t leave the house, I do almost nothing. No, I don’t need this exercise. I’m... I’m fine, I have nothing. There’s no catch, no, I don’t feel I have to do it.*
		Fatalism	Juliette: *Honestly, at the beginning [of the activity] I didn’t have any hope. I didn’t know if I was going to get there, but ... it has to be. I have to accept everything. I didn’t know if I would be able to do it, because I can’t sit down and stand up because of my knee, right? That was what I thought*.
			Interviewer: *How are you now, Mrs. Juliette?*
			Juliette: *I’m going. I’m going. That’s life.*
		Fear of worse outcome	*I did very little. For example, now I’m not feeling anything in my knee. But if it [the activity] starts to disturb it, then I start, I already start feeling the knee. I say to myself: then I won’t push myself. My knee doesn’t swell. No, no. It only hurts. Last week, it was on Tuesday, out of nowhere, I don’t know what happened, I had a little strain here. So I prefer not to force or anything because it can get worse, then, how do I do it? I’m in my nineties, I have to be very careful not to make things worse* (Lily).
		Lack of companion	*Ohhh my dear, I already did so much, I ran, I did aerobics, I ran a lot. Me and my old man [late husband] every day, early in the morning we went for a walk. Yeah, both of us went to walk early in the morning. We woke up and didn’t even drink coffee, but once in a while we had some coffee. We walked for an hour. He sometimes sat down at the beach bench and I’d say, “it’s OK”, I had some friends who also walked there. I stayed there for an hour, walked for an hour. It was good, I felt good and it was good for my health. Then after he passed away, my husband, I didn’t walk anymore, I did nothing else. He was such a good, wonderful person. 63 years of happiness. He was my partner in everything, if he were here maybe we’d be doing it together, both of us, that exercise you passed on. He was very good, I miss him* (Acacia).
How did it go?	Obstacles	Lack of motivation	*Ah, I haven’t done the exercise. I forget ... Oh, I don’t know. It’s laziness, it’s everything, everything together. I haven’t even done it. I feel discouraged. Some days I feel like that, right? I’ll do it later. Yeah, but my laziness also gets in the way, laziness. I have to get smarter, be more alert and feel that I have to move, but then when I lie down I don’t get up for anything. It’s the same old thing, my dear, it’s laziness here, I have to throw that laziness away. The whole laziness is with me, it’s my fault, nothing else* (Carnation).
		Competing demands	*But it’s funny that the prescription medication provided by the hospital, Losartan, didn’t arrive, it looks like they are out of it. But then, today I ordered [Losartan out of pocket]. I’ll get it soon. I sent [someone] to buy it in [downtown] Rio. I still have a crazy granddaughter. It’s just that I can’t ... I’m going to worry about her for what? I won’t worry. But I always get annoyed sometimes, right? It’s a lot of stress, sometimes I forget my own life, forget about doing things. When these stressful things happen, I get pissed off. On these days, I forget to do the Sit-to-stand activity. It’s a lot of concern in the head* (Butterfly).
		Routine challenge/Discipline	*I’m fine. I did the exercise only a couple of times, it’s so much to do. I’m by myself. Sometimes I forget. I do it when I remember. I even forget about this exercise, I really forget about it. Some things I do on auto-pilot, I just do them without thinking. But this Sit-to-stand activity I’m having trouble to remember* (Flora).
		Safety concern	*It’s just how I told you, when I get up, the first thing that gets me is that I have a lot of imbalance, you know? So it’s as if I were going to fall, but I don’t, I never fell because of it, but I feel like that, a thump* (Daisy).
		Weather	*I’ve done it. I do the exercise and I walk around, today I didn’t do it because there’s too much sun, very hot. I always do it in the morning. But this week no, it’s very hot, right now, it’s very hot today. When it’s hot, it’s not possible* (Carnation).

### To join, or not to join: that is the question

*Structural aspects*

The structural aspects were related to the elements of the Sit-to-stand activity. The participants usually considered the proposed activity offered to them because they were allowed flexibility to schedule it according to their pre-established routine. Another factor for those who expressed interest in pursuing the activity was the excitement of engaging in something new. Usually, these participants were retired and their lives had a slower pace than before. Sonata, for instance, portrayed it quite well. She spent her days by herself. She worked her entire life outside her home but, after she retired, her life changed. While she lived with her daughter, her child went out early in the morning and came back home late in the evening. According to her, she could benefit from some changes here and there.

On the other hand, a few participants hesitated to join the activity due to the changes it could impose on their previously established routine. The pre-established daily rituals were meaningful to participants and suggesting a change in the routine was a source of discomfort for some participants. Another pertinent concern raised by some participants was their fear of doing the activity by themselves. Overall, those who were alone during the day had a poor perception of their health or belonged to the oldest old age group and stated that having someone to do the activity with was an important element.

*Individual influence*

Factors within individuals affecting their perceptions of the Sit-to-stand activity, including ways of thinking, feeling, and acting when they were invited to do the Sit-to-stand activity are described here. When the participants perceived themselves as having physical limitations, they were more resistant to engage in the Sit-to-stand activity. However, it was not only the participants’ physical limitations and health conditions that permeated their assumptions about the Sit-to-stand activity, but also the perceived stigmatization of exercises targeting older people.

Referrals from healthcare providers also influenced the participants’ intention to perform the Sit-to-stand activity. When healthcare providers from the Family Health Strategy had previously talked to them about performing some physical activity or the benefits of the Sit-to-stand activity, they were more inclined to join it. Furthermore, the participants’ families also played an important role in their intention to perform the Sit-to-stand activity.

In addition, when the participants had goals, they agreed to participate in the activity more promptly. For instance, Juliette was housebound and her daughter and son-in-law assisted her with some activities of daily living. After she started doing the Sit-to-stand activity, she stated feeling less pain in her legs. Although the goal of the activity proposed is not to reduce pain but to strengthen muscles and assist with balance, her own perceptions and goals exerted a positive effect on her engagement in the Sit-to-stand activity.

### How did it go?

During the weekly follow-up visits, the participants and the researcher talked about the Sit-to-stand activity, their progress and their impressions of it. They also discussed whether there were any unexpected events from the previous week.

*Drivers*

The participants highlighted various elements of the Sit-to-stand activity that sustained their participation. Its low cost, translated into no gym membership fees and no specialized equipment or travel costs required; these factors were perceived as advantageous for the participants. Another positive component of the Sit-to-stand activity was the possibility of doing it in the participants’ own home, which offered them some privacy to perform the activity without feeling judged or observed.

In addition, the participants emphasized how the activity made them feel. When they could envision or feel an improvement in their mental or physical health, they seemed more willing to keep performing the Sit-to-stand activity. The easiness of the activity and the participants’ ability to adapt the activity to their reality, coupled with the opportunity of performing the Sit-to-stand activity at their own pace, also exerted a positive influence on adherence to the activity. Another important element was the participants’ sense of achievement. When they felt that they crossed self-imposed milestones, they were more confident about the future.

Having a supportive system also influenced the participants’ engagement in the activity. In most instances, they lived with family members and these relatives were quite supportive of their engagement in the Sit-to-stand activity. The family members would assist the participants when they needed help to accomplish the activity. They would encourage and remind them of the activity. Support from the participants’ social circles was also an important ingredient in their positive experience of the activity. However, the influence of supportive networks extended beyond the participants’ friends and family. A supportive healthcare system also determined their positive experience with the Sit-to-stand activity. When the participants felt that they were well cared for by the health professionals and well served by the healthcare system, they were more prone to engage in the Sit-to-stand activity. Finally, the participants also had an easier time when they were able to fit the activity into their daily routine.

*Obstacles*

The participants highlighted various elements of the Sit-to-stand activity that hindered their participation. Many of them related the impact of their present health conditions and their symptoms such as lack of balance, lack of strength, fatigue and pain as factors hindering their ability to do the Sit-to-stand activity. At times, they did not perceive a need to perform the activity. The participants who were not able to visualize the benefits of the activity engaged in it less frequently. In addition, those who believed that, regardless of their actions or deeds, there are some things in life that are predestined to occur, had more difficulties engaging in the activity. This sort of fatalism was illustrated by some participants while doing the Sit-to-stand activity. Likewise, when the participants were fearful of reaching a worse outcome, they had more difficulties when engaging in the activity.

Another recurrent complaint was lack of companion to do the activity. Most of participants lived with or close to their family members but a few of them spent the day by themselves due to their family members’ work schedules during the day or their significant other not being present. For instance, in one of our informal interviews, Acacia conveyed that her late husband was her companion for travel and in physical activities but that, since his passing away, she had lost the person who used to do activities with her.

Some participants mentioned that lack of motivation, safety concerns, weather and competing demands in their lives precluded them from trying to perform the activity. The issues varied from family problems to health concerns. Yet, one of the most cited reasons hindering the participants’ engagement in the activity was their inability to insert the activity into their routine.

## Discussion

The key findings from this study are that the experience of mobility-challenged older people with the Sit-to-stand activity was dependent on their mobility expectations involving many factors that worked together to influence their beliefs and attitudes towards the activity, preferences, behaviors, and cultural perceptions. The participants seemed to find the activity enjoyable; however, the most noticeable shortcomings for their engagement in the Sit-to-stand activity was related to contextual factors, namely gaps in their personal and intrapersonal needs. Four key components are brought into the discussion to shed light on Brazilian older people’s experiences with the Sit-to-stand activity: (a) establishing intentions, (b) the experience with the components of the activity, (c) the personal experience with the activity, and (d) the interpersonal experience with the activity.

### Establishing intentions

Before engaging in the Sit-to-stand activity, the participants referred to perceived barriers and benefits of doing so. Changes in their routine and the perceived need to have someone to do the Sit-to-stand activity with were observed as obstacles to engaging in it. However, having flexibility to fit the activity into the participants’ preferred times and the opportunity of trying something new were perceived as catalysts to their engagement in the Sit-to-stand activity. This information will help us to consider how the many elements of the activity itself may have influenced the adoption process. This knowledge has important applications as it informs us about the Sit-to-stand activity potential areas for refinement in the knowledge translation process.

A few participants stated having or suffering negative stigmatization of growing old and exercising. There is mounting evidence to suggest that older people constitute a stigmatized group around the world^39^. In fact, “ageism” reveals the stigma and negative attitudes associated with advanced age that are linked to mental and physical health consequences, including less desire to live a healthy lifestyle^40^ ^-^ ^41^. This is corroborated by the literature on the stereotype threat, suggesting that stigmatized individuals avoid the negative experience of such threat by disengaging from important activities^42^. The issue of ageism is so rampant that the UN Decade of Healthy Ageing presents an agenda with calls to action, where the first action is to change how people think, feel and act toward age and ageing^13^. It is argued that reducing ageism is important given its widespread impact on the way issues faced by older people are seen, priorities are evaluated, and solutions are selected. Ageism relegates older people to the sidelines, lowering appreciation of their social capital and restricting their access to services.

Finally, showing intention to engage in the Sit-to-stand activity and having goals were perceived as facilitators to engagement in the Sit-to-stand activity. Both of these factors are aligned to several social psychological models, including the theory of reasoned action^(43-44^), Triandis^45^ attitude-behavior theory, and the protection motivation theory^46^. These theories concur with the proposal that the most immediate and important predictor of a person’s behavior is his/her intention to perform it and having goals.

### The experience with the components of the activity

Identifying the structural elements of a future intervention is a crucial part of unpacking the “intervention black box”. Knowledge of individuals’ perceptions of structural elements can be used to identify specific practices that promote adoption and optimize interventions. During and after engaging in the Sit-to-stand activity, the participants of this study reported the low cost for this activity for being home-based, the possibility to do it at their own pace, and its ease of integration into a daily routine as components of the activity that supported its adoption. However, it is also important to consider other components of the activity that undermined its adoption, such as safety concerns and lack of a companion.

The results of this study indicate that there were a range of factors that contributed to the participants’ engagement in the Sit-to-stand activity. The activity’s low-cost was attributed to non-requirement of specialized equipment and travel, which, in turn, is connected to the activity’s accessibility. This result was built on a meta-synthesis of qualitative studies of independently living older people’s (+65 years old) experiences of physical activity interventions in non-clinical contexts and found that keeping costs to a minimum was important, as many aged individuals earn limited incomes^47^.

In this study, some of the participants described the home-based scope of the Sit-to-stand activity as meeting their needs, particularly because it allowed them to enjoy their privacy while performing the activity. This result is in line with previous researchers who also found that doing exercises at their home allowed the participants the privacy to perform them without feeling judged or observed^48^. On the other hand, a few participants felt that the home-based scope of the activity was disadvantageous because it removed from them the opportunity of social integration. However, it has been suggested that, while group-based programs are more effective in the short term, home-based programs appear to be more effective when it comes to physical activity maintenance in older individuals^49^.

For these participants, the possibility of doing the Sit-to-stand activity at their own pace, its adaptability, and its ease of integration into their daily routine worked as facilitators. This result is also consistent with previous research findings pointing out that “older adults should be encouraged to go at their own pace, although they should also be supported to increase intensity or duration when they are able to do more^47^. This is important, as they may have self-limiting expectations that have to be addressed but that need to be handled sensitively”. In addition, the ability to integrate the Sit-to-stand activity into participants’ daily routine was previously mentioned in other studies as a driver to engagement in the activity (indicating that a collaborative and mindful approach to physical activity seems more acceptable to older people)^50^.

### The personal experience with the activity

Perceived health benefits, having goals, being motivated, and having a sense of achievement were individual factors facilitating the Sit-to-stand activity. Conversely, the impact of comorbidities, fatalism, no perceived need, fear of worse outcomes, and lack of motivation were individual factors hindering the activity. A key facilitator for performing the Sit-to-stand activity was the participants’ observations of personal benefits because of the activity. That was possible when they set small achievable goals, which, in turn, motivated and gave them a sense of achievement. The role of experiencing some personal benefit as a result of the activity was previously discussed in studies with older people^51^ ^-^ ^52^. The authors found that higher perceived health benefits and greater self-efficacy were associated with physical activity among older people. Thus, the potential benefits of engaging in a lifestyle that incorporates regular physical activity are to improve physical function, independence and quality of life^52^. These changes have been shown to be beneficial even when physical activity is started at a later stage in life^52^.

In a reverse situation, the participants’ comorbidities, fears of worse outcomes, lack of motivation, fatalistic views and no perceived need were key barriers to performing the Sit-to-stand activity. Although having comorbidities was probably the most often mentioned barrier to engaging in the Sit-to-stand activity among the participants, perceived health benefits from engaging in the activity were the most often reported motivators for the activity. This finding is consistent with the literature, where it is revealed that perceived health benefits represent a factor that can work in both directions^41^. They can be viewed as a facilitator motivating physical activity or as a barrier detracting from physical activity when no benefit is anticipated.

In addition, when these study participants had set goals for themselves, it fostered their participation in the Sit-to-stand activity. Goals are defined as internal representations of desired outcomes, events or processes. Goal setting is one of the most widely applied and universally accepted strategies used to increase physical activity^53^.

Over time, satisfactory feelings such as motivation and achievement appeared to exert a positive influence on the participants’ engagement in the Sit-to-stand activity. Other researchers have shown that similar remarks predict higher levels of adherence to physical activity and exercise^47^ ^,^ ^54^ ^-^ ^56^. In addition, the individual’s motivation is a key factor influencing mobilization, especially if they are able to safely walk by themselves or with family members^56^.

### The interpersonal experience with the activity

The participants included in this study described having a supportive system and a companion, and not having competing demands as interpersonal factors influencing their experience with the Sit-to-stand activity. Family and friends played a role in encouraging participation in, and adherence to, the Sit-to-stand activity. Both the importance of concrete support, such as being able to push and help the participants, as well as emotional support was emphasized. Others researchers have shown that support from family, friends, peers and caregivers is considered critical to promoting and maintaining engagement with any exercise/activity intervention^48^ ^,^ ^57^. The participants’ choice in accepting engagement in a physical activity is framed by the physiological and psychological impacts of the intervention and also by the social and cultural structures in which a person is living^58^. Thus, social and cultural factors appear to shape expectations of engagement in the Sit-to-stand activity.

The meaning of social networks was also shown by feelings of obligation in relation to the family. Perceived obligations to the family resulted in competing demands hindering the participants’ engagement in the Sit-to-stand activity. Difficulties balancing and prioritizing important factors in life and lack of support from the family have been found to arouse feelings of guilt and duty, often resulting in decreased adherence and dropout from physical activities^59^. It is noteworthy that, in numerous studies, a common barrier reported by underprivileged groups of older people was competing family responsibilities^59^.

This study is the first one of its kind to capture perceptions and experiences of the Sit-to-stand activity in urban Brazilian community-dwelling older people. As other researchers begin to evaluate populations of older people with mobility challenges from countries in the Global South and those managing multiple comorbidities, data from this study may be used as a means of comparison. The findings of this study are rich because there was wide variation among the participants’ demographic characteristics. Thus, this study may assist in understanding how factors related to older people’ cultural, psychosocial, physical and health environments facilitate or hinder appropriateness and usefulness of interventions related to physical activity in their daily lives. Findings from this study also support the International Classification of Functioning, Disability and Health for understanding mobility within the functioning - disability continuum.

Functional mobility is vital for activity and participation and reduces dependence. Specifically in the Global South, older people with significant mobility challenges are at risk for additional mobility-related poor health outcomes^11^ ^-^ ^12^. Nurses are in a prime position to facilitate changes in practice and education that could reduce disability, improve mobility and increase trust between patients and healthcare providers. Findings from this study may be used to improve older people’s health, as they provide important clues about how to improve strategies used by nurses to assist older people in effectively managing mobility challenges.

Recognizing and understanding perceived barriers to mobility in older people is an important first step toward developing clinical awareness and minimizing mobility challenges. In Brazil, among the Family Health Strategy team tasks, home visits are one of the pillars of the program. They allow healthcare providers to know the social context and identify the patients’ health needs to support actions aimed at preventing diseases and promoting community health. Thus, an important finding of this study was that nurses were one of the professionals of the healthcare team who frequently performed home visits. These community health nurses from the Family Health Strategy program had increased access to the patients’ home environment, which allowed for frequent observations of their context. The access enjoyed by nurses to the older people’s context offers them the opportunity to further their role as patient advocates.

Additionally, the holistic nature of nursing care adequately prepares nurses to serve as liaisons between patients and health services, thus facilitating integration across all care domains. Nurses should not underestimate their ability to influence access to appropriate, efficient, and effective quality care. They are in an excellent position to share with various constituencies the importance of appropriate health services available.

The current study suggests the need for producing more research on cultural preferences and norms regarding physical activity facilitators and barriers in the Global South. Older people’s cultural values and norms regarding physical activity behaviors are paramount to implement the Sit-to-stand activity as an intervention, which suggests that future research on marginalized and stigmatized groups needs to avoid lumping different ethno-cultural groups together in order to better understand the influence of distinct cultures on behavior. Thus, taking into consideration these findings, we believe that a full feasibility study should be undertaken now that the cultural, social, physical and health environments affecting older people’s mobility and home-based physical activity are better known.

Despite this study’s valuable findings concerning older people’ perceptions and experiences with the Sit-to-stand activity, there are limitations as with any research study. Recruitment was limited to older people living with mobility challenges who consented to participate. The perceptions and experiences of older people who refused to participate may differ from those who willingly participated. The Hawthorne effect might have influenced this study, for we performed participant observation and, upon the participants’ request, during some interactions between them and the researcher, the participants’ children, other family members or caretakers were present^60^. In addition, due to limited time and resources, the perceptions and experiences of healthcare providers, family members and policy makers were not examined. Further research is necessary into other stakeholders’ perceptions of the Sit-to-stand activity, and whether there are shared perceptions.

## Conclusion

This study provides new information to the field of inquiry on mobility of older people because it focuses on the perceptions of a specific age group, and attends to the interrelationships among health conditions and contextual factors affecting the mobility of older people in Brazil. As the population ages, it becomes increasingly imperative to understand older people’ perceptions and experiences. By eliciting the perspectives of older people with this innovative Sit-to-stand intervention in countries from the Global South, this study contributes a much-needed window into individual understandings, expectations of, and self-reported reasons for uptake in or lack of engagement with the intervention. Establishment of intentions, knowledge of personal and interpersonal experiences, as well as the experience with the components of the intervention, were key considerations for older people’s uptake and engagement. Older people will likely continue to require differentiated care, where interventions that better address the needs of this population segment are taken into consideration. These novel findings, aggregating older people’s perspectives, represent the first step to addressing the needs of this population group.
